# Modelling the effects of combining pre-erythrocytic vaccines against *Plasmodium falciparum*

**DOI:** 10.1186/1475-2875-13-S1-P90

**Published:** 2014-09-22

**Authors:** Andrew Walker, Sunetra Gupta

**Affiliations:** 1 Department of Zoology, University of Oxford, Oxford, UK

## Background

A high efficacy multi-stage multi-epitope malaria vaccine would be an invaluable weapon in the eradication of malaria. Currently, the best progress in subunit vaccine research has been seen in targeting pre-erythrocytic (PE) stages of the parasite. In phase IIa trials, RTS,S in adjuvant was capable of inducing sterile protection in 50% of individuals [1], and ME-TRAP in a heterologous prime-boost regime saw around 21% efficacy [2]. Here we present a mathematical model investigating the effects of combining these two approaches.

## Materials and methods

A mathematical model was developed to describe *Plasmodium falciparum* parasite dynamics within an immunonaive host. The model was parameterised using data from non-vaccine recipients in Controlled Human Malaria Infection (CHMI) studies, and then used to study the effects of vaccine induced immune responses against the sporozoite and hepatocytic stages of the life cycle, and the effects of combining them.

## Results

Using data from phase IIa trials of RTS,S [1]and ME-TRAP [2], we predict that near total sterile protection can be achieved by combining these vaccines. Even very low efficacy (<1%) vaccines are shown to combine to confer sterile protection in up to 43% of individuals.

## Conclusions

We find a heterologous vaccine treatment to act synergistically: the efficacy of the combination far outweighs the sum of its parts. This suggests that a high efficacy multi-stage vaccine may well be within reach, and may utilise some of the tools presently available.
